# Relative sea-level data from the SEAMIS database compared to ICE-5G model predictions of glacial isostatic adjustment

**DOI:** 10.1016/j.dib.2019.104600

**Published:** 2019-10-03

**Authors:** Thomas Mann, Maren Bender, Thomas Lorscheid, Paolo Stocchi, Matteo Vacchi, Adam Switzer, Alessio Rovere

**Affiliations:** a Leibniz Centre for Tropical Marine Research (ZMT), Fahrenheitstraße 6, 28359 Bremen, Germany; b University of Bremen, Center for Marine Environmental Sciences (MARUM), Leobener Straße 8, 28359 Bremen, Germany; c NIOZ Royal Netherlands Institute for Sea Research, Department of Coastal Systems, Utrecht University, Den Horn, Texel, the Netherlands; d Geography, College of Life and Environmental Sciences, University of Exeter, EX4 4RJ, Exeter, UK; e Asian School of the Environment, Nanyang Technological University, 649812, Singapore; f Earth Observatory of Singapore, Nanyang Technological University, 649812, Singapore

**Keywords:** Glacial isostatic adjustment, Sea-level highstand, Sea-level rise, Climate change, Tectonics

## Abstract

The SEAMIS database (Mendeley data repository; https://doi.org/10.17632/wp4ctb4667.1) contains 546 relative sea-level indicators from 31 different studies within the broader Southeast Asian region including the Maldives, India and Sri Lanka. Here we compare quality-controlled and site-specific relative sea-level data from 23 studies from the SEAMIS database to a suite of ICE-5G glacial isostatic adjustment models. The relation between robust and, if applicable, tectonically corrected relative sea-level data with the broad predictions of glacial isostatic adjustment models is interpreted and discussed in the article “Holocene sea levels in Southeast Asia, Maldives, India and Sri Lanka: The SEAMIS database” [1] in Quaternary Science Reviews.

**Table:** Specifications Table

Subject area	*Earth Sciences*
More specific subject area	*Coastal geomorphology*
Type of data	*Tables, graphs, figures, netCDF files*
How data was acquired	*Standardization of published data; modeling*
Data format	*Published RSL data: standardized and quality-controlled; netCDF files of modeled RSL: raw*
Experimental factors	*Data considered originate from previous studies carried out in Southeast Asia, Maldives, India and Sri Lanka and contain Holocene RSL information*
Experimental features	*Data were collected from literature review*
Data source location	*Southeast Asia, Maldives, India and Sri Lanka*
Data accessibility	*SEAMIS database and updates, netCDF files of ICE-5G model output and MATLAB script to plot data at*https://github.com/Alerovere/SEAMIS*; SEAMIS database containing RSL indicators also at*https://doi.org/10.17632/wp4ctb4667.1

**Table:** 

**Value of the Data** • Data are useful to calibrate earth- and ice-models in glacial isostatic adjustment simulations • Data is beneficial for modelers of glacial isostatic adjustment processes and field geologists in Southeast Asia • Data can be easily updated by other researchers and compared to other models of glacial isostatic adjustment • Data allow an evaluation of potential post-formational changes in the elevations of relative sea-level markers • Data allow a validation of model parameters

## 1 Data

The dataset (i.e. the SEAMIS database as of July 2019) comprises 546 Holocene relative sea-level indicators for Southeast Asia and surrounding regions (https://github.com/Alerovere/SEAMIS, https://doi.org/10.17632/wp4ctb4667.1, [1]). Age-elevation information of published relative sea-level data have been transformed into comparable relative sea-level indicators using a standardized protocol [2]. Quality-controlled, site-specific relative sea-level indicators are here compared to modeled relative sea-level change at each site generated with the ICE-5G geophysical model (Table 1, [3]).

**Table 1:** Details on the Earth model parameters and different mantle viscosity profiles employed to simulate glacial isostatic adjustment in combination with the Ice model ICE-5G in the areas of interest. Model short names refer to the different model curves on Fig. 1, Fig. 2, Fig. 3, Fig. 4, Fig. 5, Fig. 6, Fig. 7, Fig. 8, Fig. 9, Fig. 10, Fig. 11, Fig. 12, Fig. 13, Fig. 14, Fig. 15, Fig. 16, Fig. 17, Fig. 18, Fig. 19, Fig. 20, Fig. 21, Fig. 22.

Model short name	Ice model	Earth model parameters
ice5g-vm2-90km.nc	ICE-5G	Upper Mantle = 0.25 × 10^21^ Pa•s Transition Zone = 0.5 × 10^21^ Pa•s Lower Mantle = 5 × 10^21^ Pa•s Lithosphere Thickness = 90 km
ice5g-vm2b-90km.nc	ICE-5G	Upper Mantle = 0.25 × 10^21^ Pa•s Transition Zone = 0.25 × 10^21^ Pa•s Lower Mantle = 5 × 10^21^ Pa•s Lithosphere Thickness = 90 km
ice5g-vm2-120km.nc	ICE-5G	Upper Mantle = 0.25 × 10^21^ Pa•s Transition Zone = 0.5 × 10^21^ Pa•s Lower Mantle = 5 × 10^21^ Pa•s Lithosphere Thickness = 120 km
ice5g-vm3-90km.nc	ICE-5G	Upper Mantle = 0.25 × 10^21^ Pa•s Transition Zone = 0.5 × 10^21^ Pa•s Lower Mantle = 10 × 10^21^ Pa•s Lithosphere Thickness = 90 km
ice5g-vm4-90km.nc	ICE-5G	Upper Mantle = 0.25 × 10^21^ Pa•s Transition Zone = 0.5 × 10^21^ Pa•s Lower Mantle = 100 × 10^21^ Pa•s Lithosphere Thickness = 90 km

The present dataset comprises a collection of RSL data from 23 studies that have been conducted in 22 locations. Fig. 1, Fig. 2, Fig. 3, Fig. 4, Fig. 5, Fig. 6, Fig. 7, Fig. 8, Fig. 9, Fig. 10, Fig. 11, Fig. 12, Fig. 13, Fig. 14, Fig. 15, Fig. 16, Fig. 17, Fig. 18, Fig. 19, Fig. 20, Fig. 21, Fig. 22 present site-specific, standardized, quality-controlled and, if possible (see Ref. [1]), tectonically corrected age-elevation information of relative sea-level indicators together with the modeled relative sea level.

**Fig. 1:**
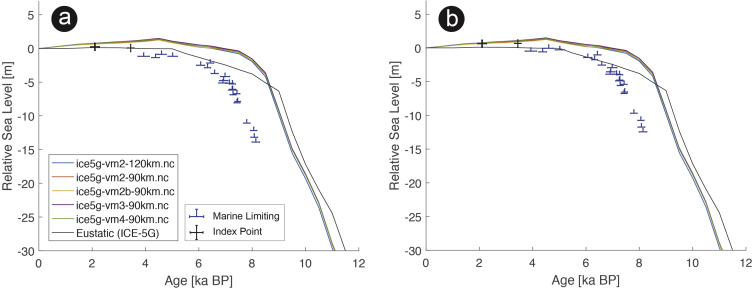
Standardized Holocene relative sea-level data obtained from Ref. [4] in comparison to glacial isostatic adjustment geophysical model predictions for South Maalhosmadulu Atoll, Maldives. a) Original sample elevations are shown. b) Data corrected for subsidence based on a number of constraints regarding the timing and elevation of Last interglacial sea level and the magnitude of karstification resulting from subaerial exposure of the Last interglacial reef carbonate during the glacial (see Ref. [1] and above).

**Fig. 2:**
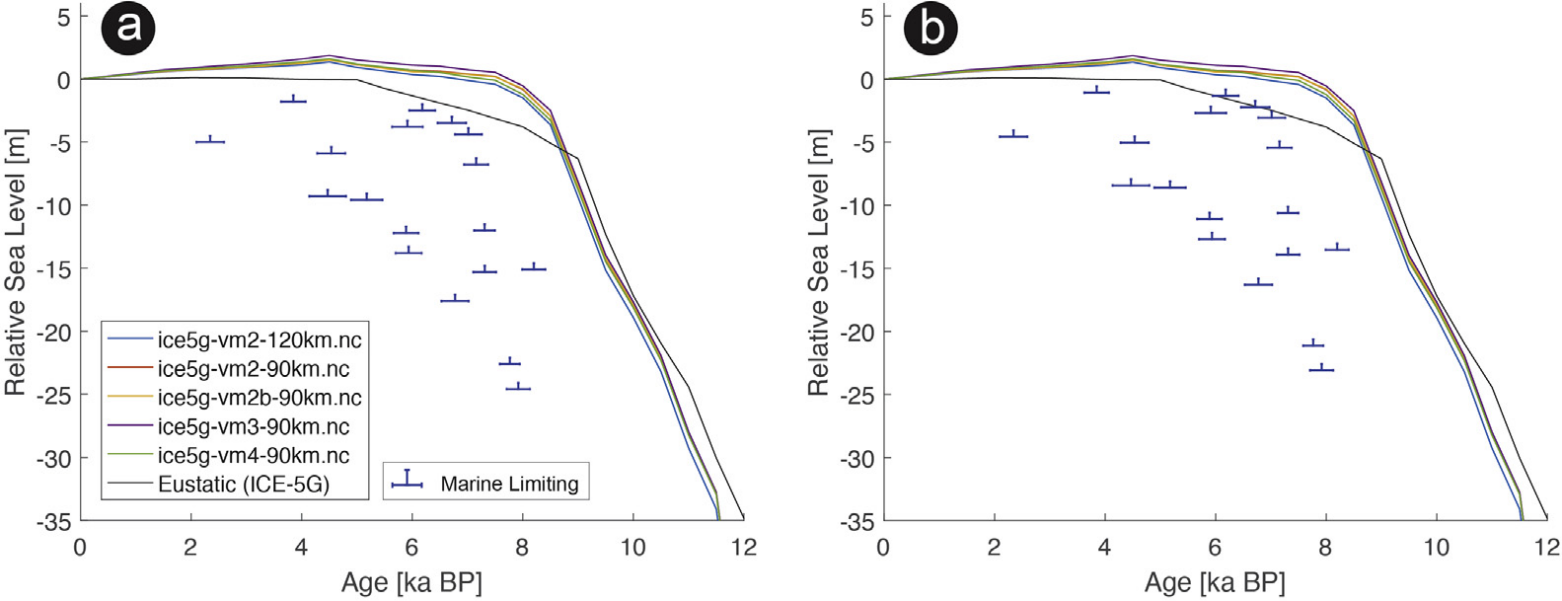
Standardized Holocene relative sea-level data obtained from Ref. [5] in comparison to glacial isostatic adjustment geophysical model predictions for Palau Islands in the western Pacific. a) Original sample elevations are shown. b) Data corrected for subsidence based on a number of constraints regarding the timing and elevation of Last interglacial sea level and the magnitude of karstification resulting from subaerial exposure of the Last interglacial reef carbonate during the glacial (see Ref. [1] and above).

**Fig. 3:**
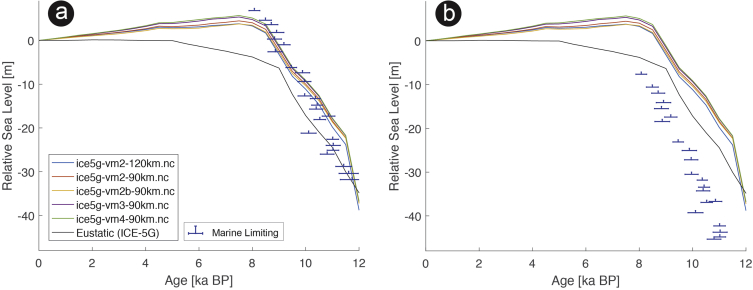
Standardized Holocene relative sea-level data obtained from Ref. [6] in comparison to glacial isostatic adjustment geophysical model predictions for Huon Peninsula, Papua New Guinea. a) Original sample elevations are shown. b) Data corrected for tectonic uplift based on a number of constraints regarding the timing and elevation of Last interglacial sea level and the magnitude of karstification resulting from subaerial exposure of the Last interglacial reef carbonate during the glacial (see Ref. [1] and above).

**Fig. 4:**
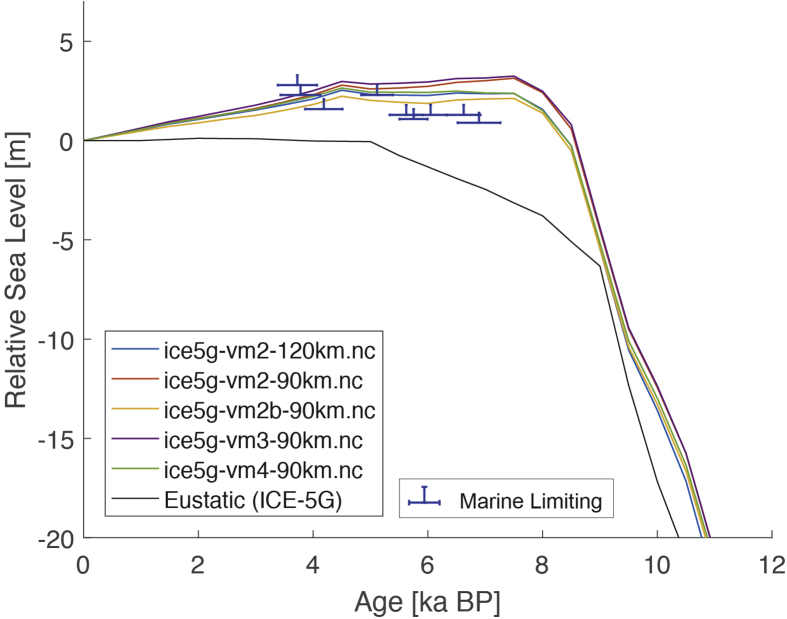
Standardized Holocene relative sea-level data obtained from Ref. [7] in comparison to glacial isostatic adjustment geophysical model predictions for the section between Cape Comorin and Rameswaram in Southeastern India.

**Fig. 5:**
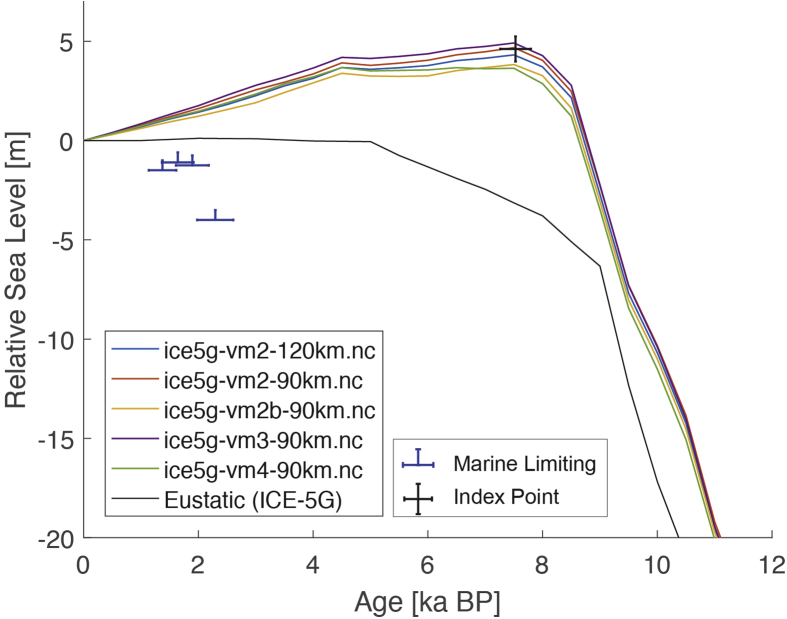
Standardized Holocene relative sea-level data obtained from Ref. [8] in comparison to glacial isostatic adjustment geophysical model predictions for the Pulicat Lagoon in Southeastern India.

**Fig. 6:**
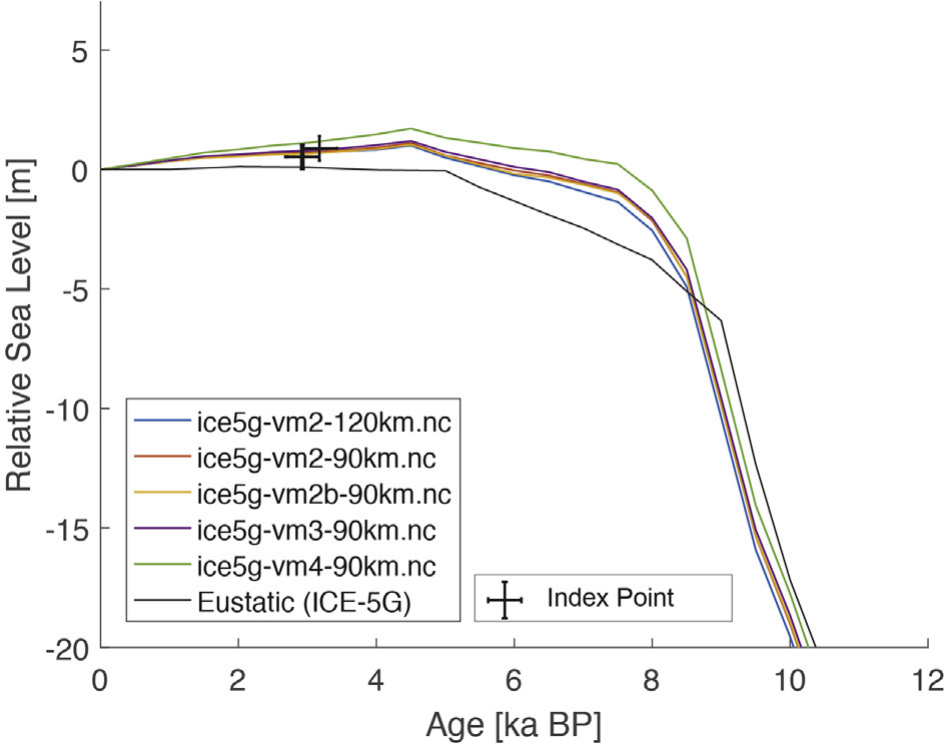
Standardized Holocene relative sea-level data obtained from Ref. [9] in comparison to glacial isostatic adjustment geophysical model predictions for the Cocos (Keeling) Islands in the eastern Indian Ocean.

**Fig. 7:**
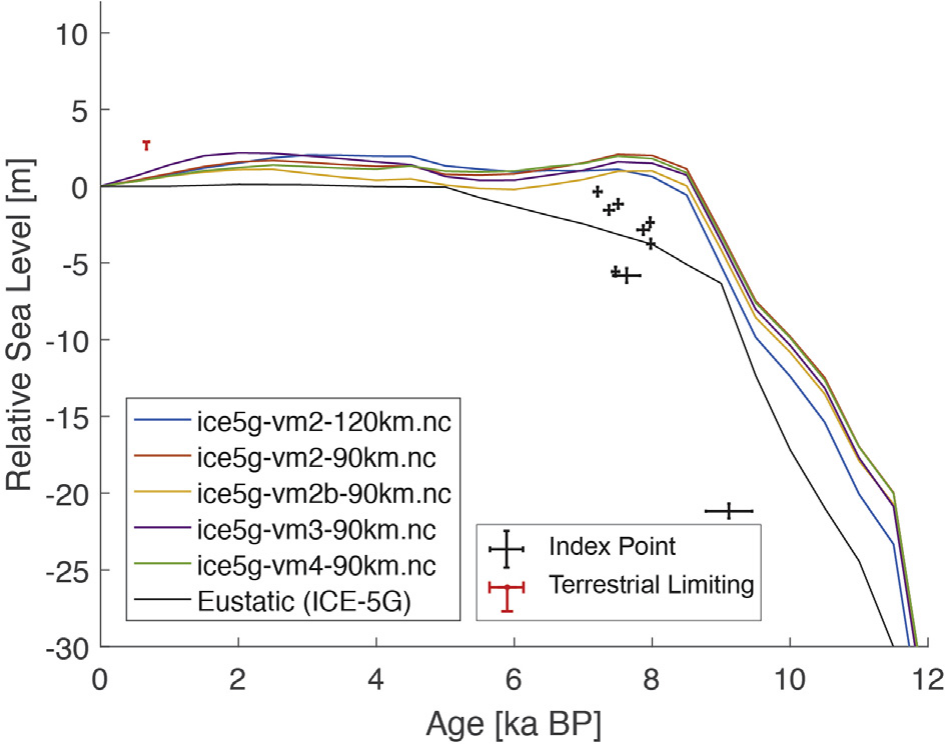
Standardized Holocene relative sea-level data obtained from Ref. [10] in comparison to glacial isostatic adjustment geophysical model predictions for the Mekong River lowland near Phnom Penh, Cambodia.

**Fig. 8:**
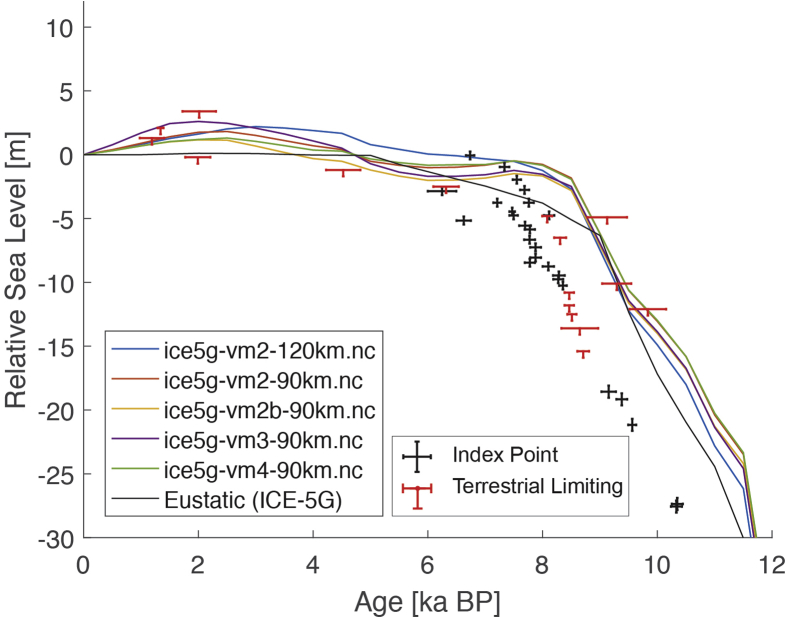
Standardized Holocene relative sea-level data obtained from Ref. [11] in comparison to glacial isostatic adjustment geophysical model predictions for the Mekong River lowland near Phnom Penh, Cambodia.

**Fig. 9:**
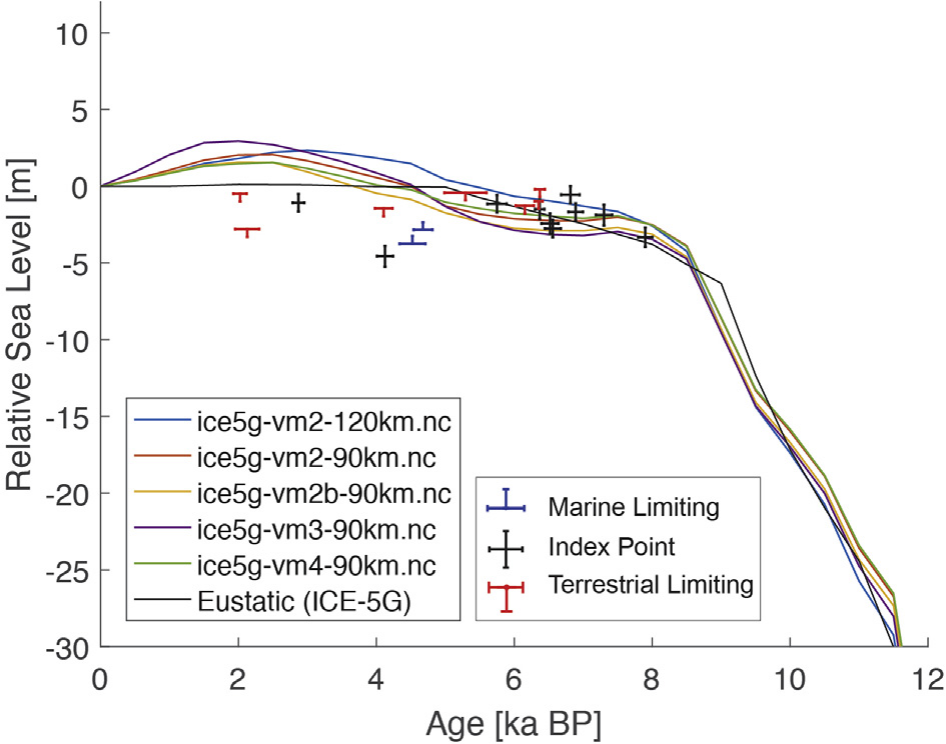
Standardized Holocene relative sea-level data obtained from Ref. [12] in comparison to glacial isostatic adjustment geophysical model predictions for the northeastern Mekong River Delta, Vietnam.

**Fig. 10:**
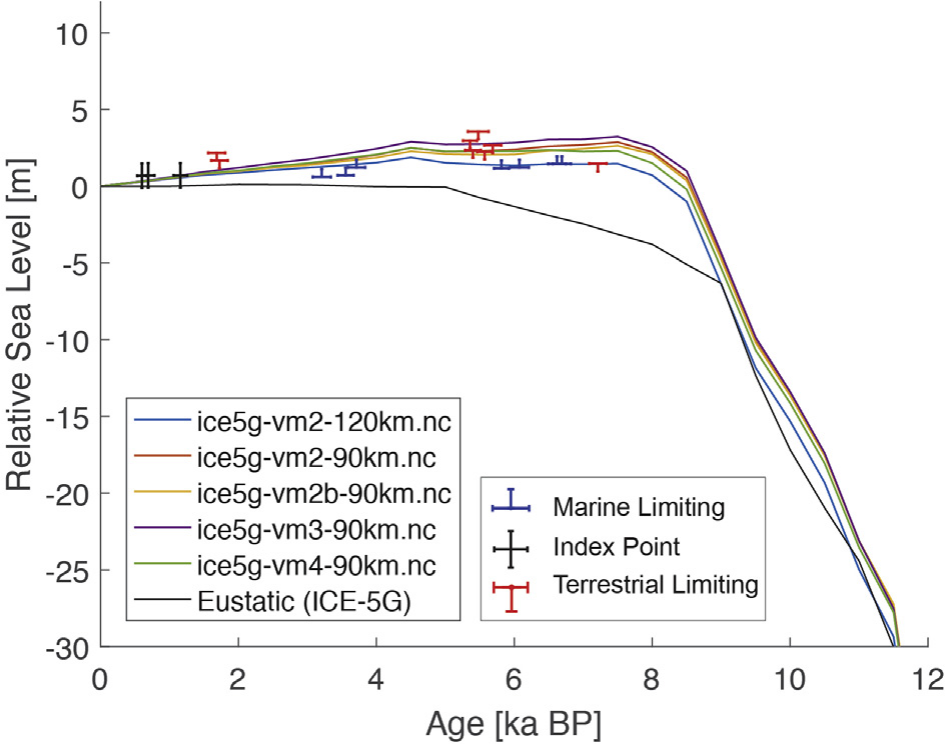
Standardized Holocene relative sea-level data obtained from Ref. [13] in comparison to glacial isostatic adjustment geophysical model predictions for the section between Cà Ná and Son Hài in southeast Vietnam.

**Fig. 11:**
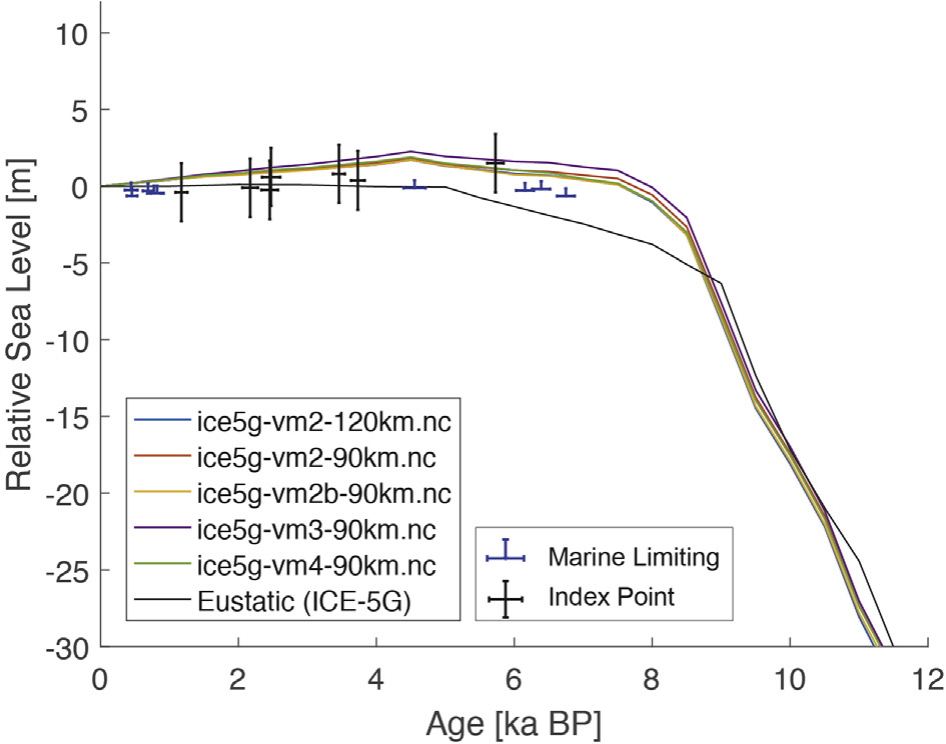
Standardized Holocene relative sea-level data obtained from Ref. [14] in comparison to glacial isostatic adjustment geophysical model predictions for the Phang-nga Province, Thailand.

**Fig. 12:**
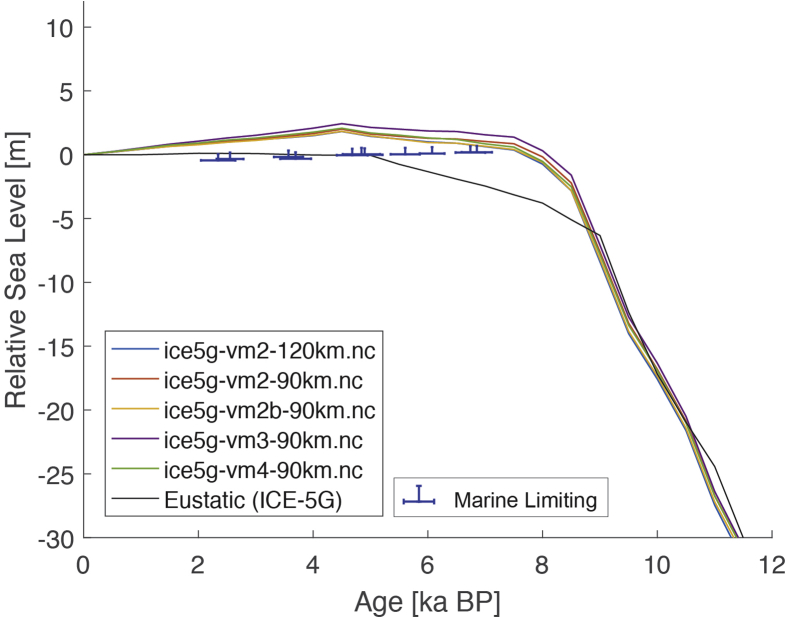
Standardized Holocene relative sea-level data obtained from Ref. [15] in comparison to glacial isostatic adjustment geophysical model predictions for Phuket, South Thailand.

**Fig. 13:**
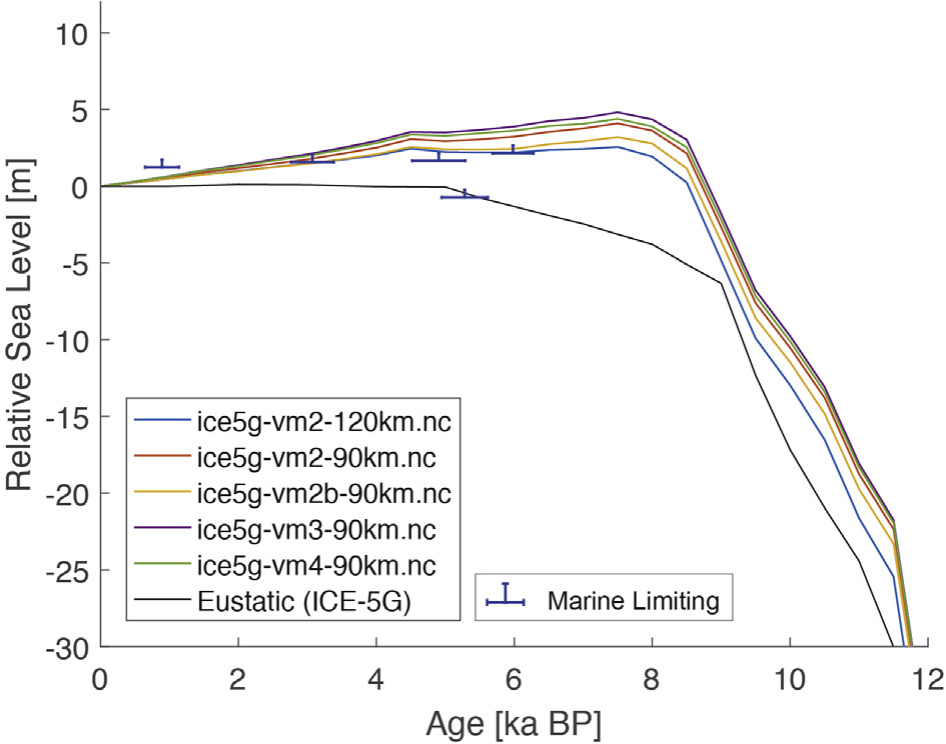
Standardized Holocene relative sea-level data obtained from Ref. [16] in comparison to glacial isostatic adjustment geophysical model predictions for the section between Langkawi and Terengganu-Pahang at the west coast of Peninsular Malaysia.

**Fig. 14:**
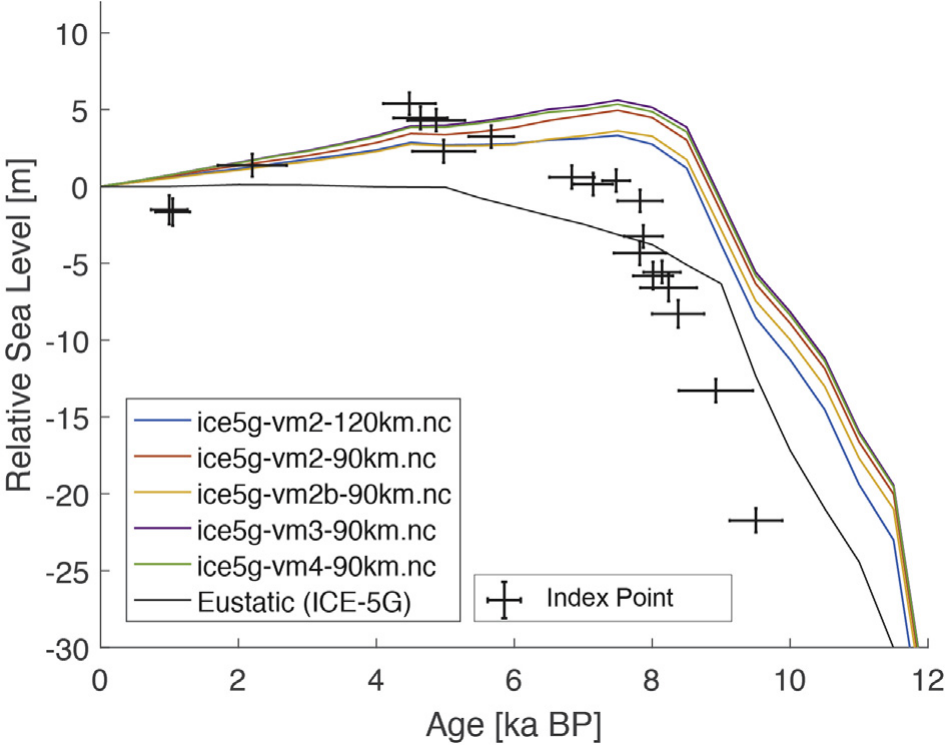
Standardized Holocene relative sea-level data obtained from Ref. [17] in comparison to glacial isostatic adjustment geophysical model predictions for the section between Port Dickinson, Malaysia and Singapore.

**Fig. 15:**
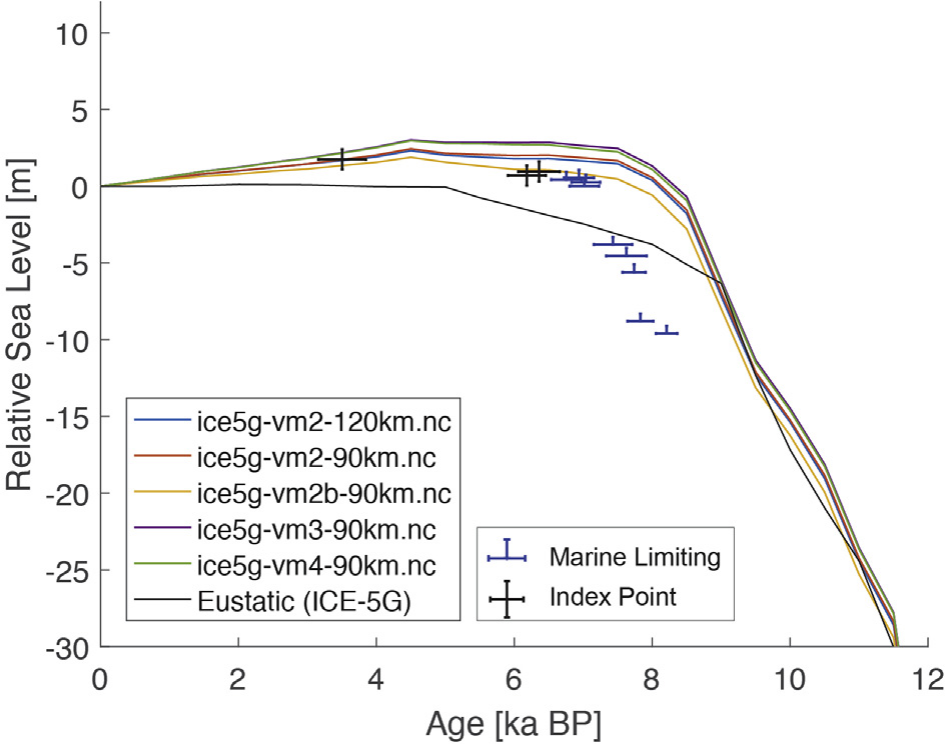
Standardized Holocene relative sea-level data obtained from Ref. [18] in comparison to glacial isostatic adjustment geophysical model predictions for the Sungei Nipah catchment, Singapore.

**Fig. 16:**
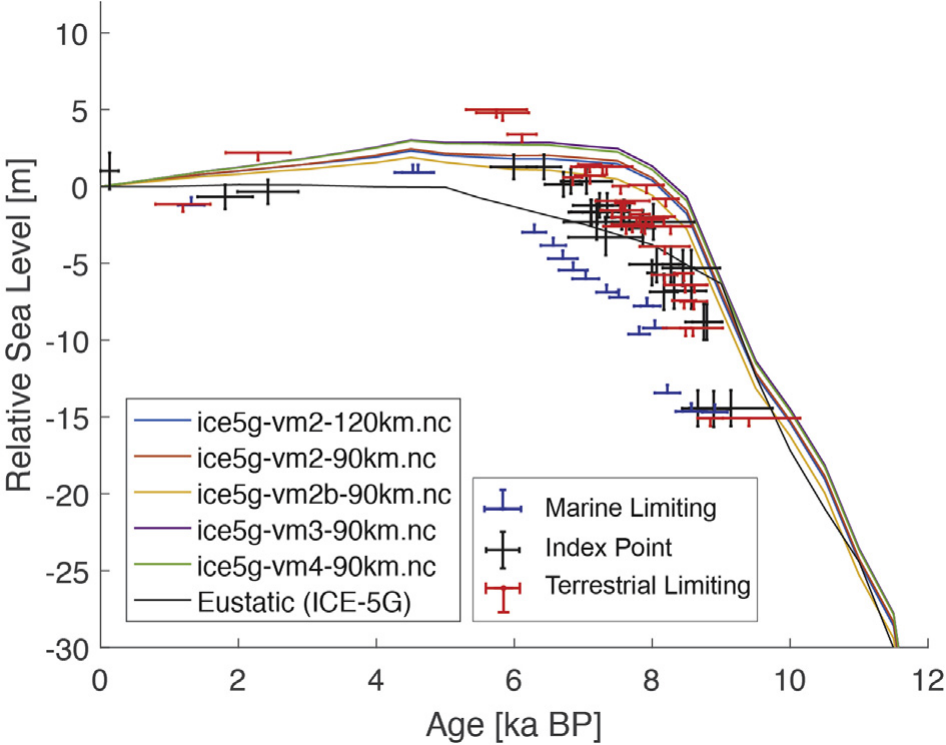
Standardized Holocene relative sea-level data obtained from Refs. [19,20] in comparison to glacial isostatic adjustment geophysical model predictions for the Geylang district, Singapore.

**Fig. 17:**
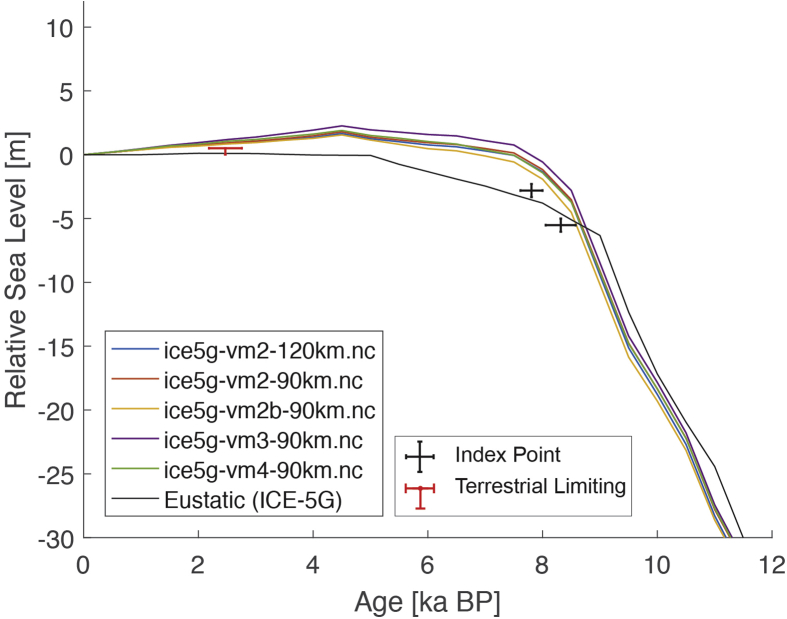
Standardized Holocene relative sea-level data obtained from Ref. [21] in comparison to glacial isostatic adjustment geophysical model predictions for the Great Songkhla Lakes, Malay-Thai Peninsula.

**Fig. 18:**
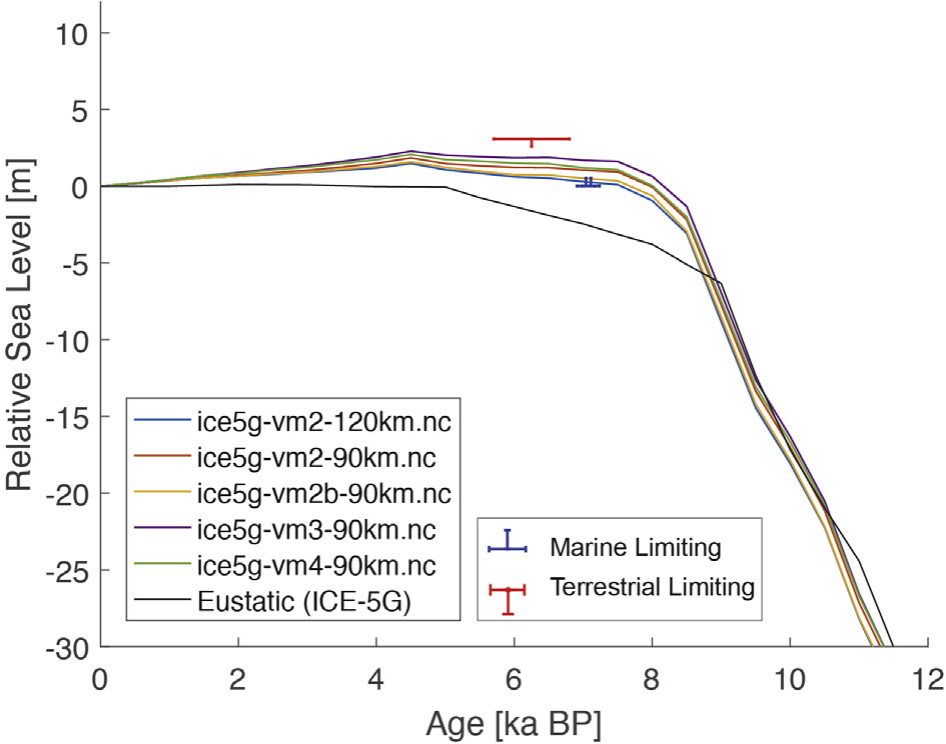
Standardized Holocene relative sea-level data obtained from Ref. [22] in comparison to glacial isostatic adjustment geophysical model predictions for the area near Merang, Malaysia.

**Fig. 19:**
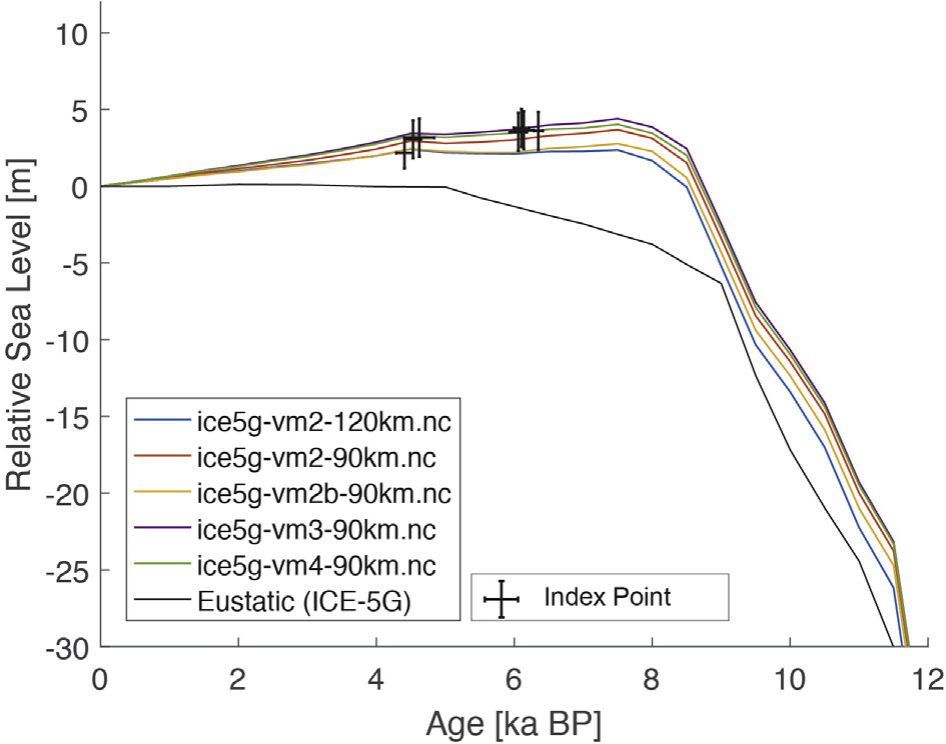
Standardized Holocene relative sea-level data obtained from Ref. [23] in comparison to glacial isostatic adjustment geophysical model predictions for Kelang and Kuantan, Peninsular Malaysia.

**Fig. 20:**
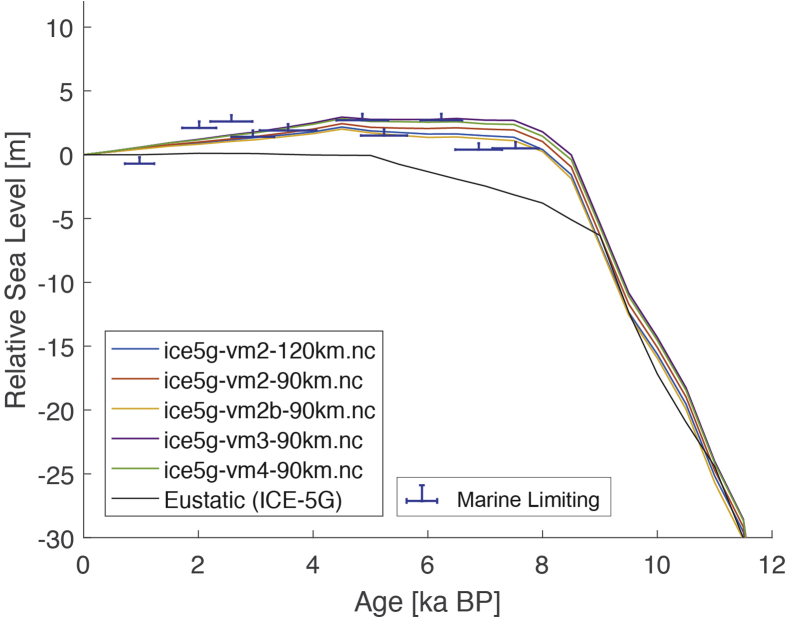
Standardized Holocene relative sea-level data obtained from Ref. [24] in comparison to glacial isostatic adjustment geophysical model predictions for Tioman Island, Malaysia.

**Fig. 21:**
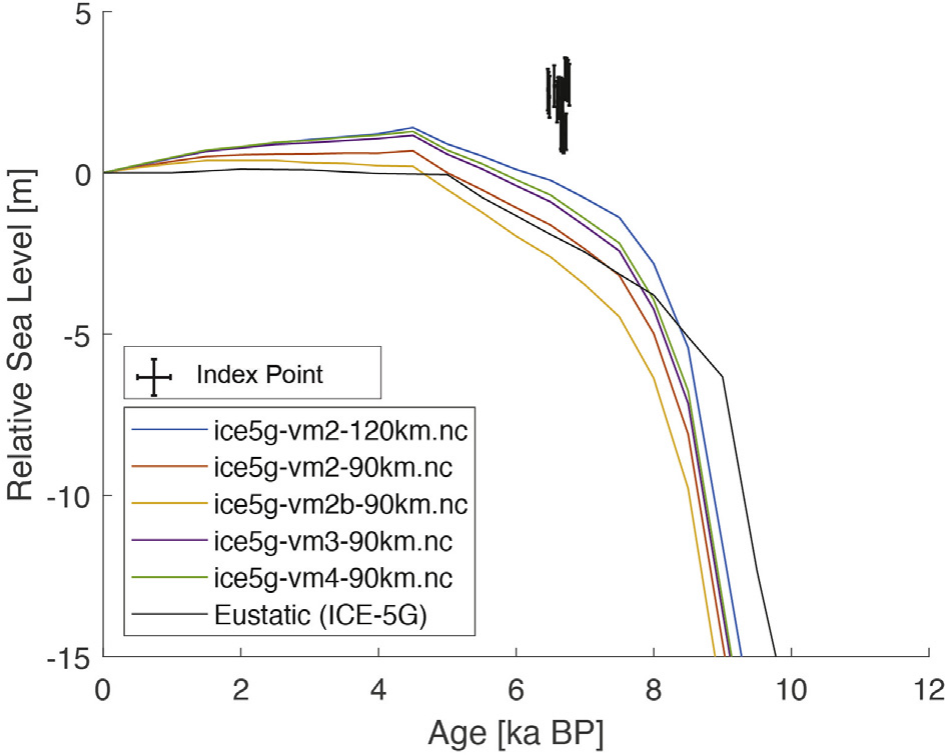
Standardized Holocene relative sea-level data obtained from Ref. [25] in comparison to glacial isostatic adjustment geophysical model predictions for the Belitung area, Indonesia.

**Fig. 22:**
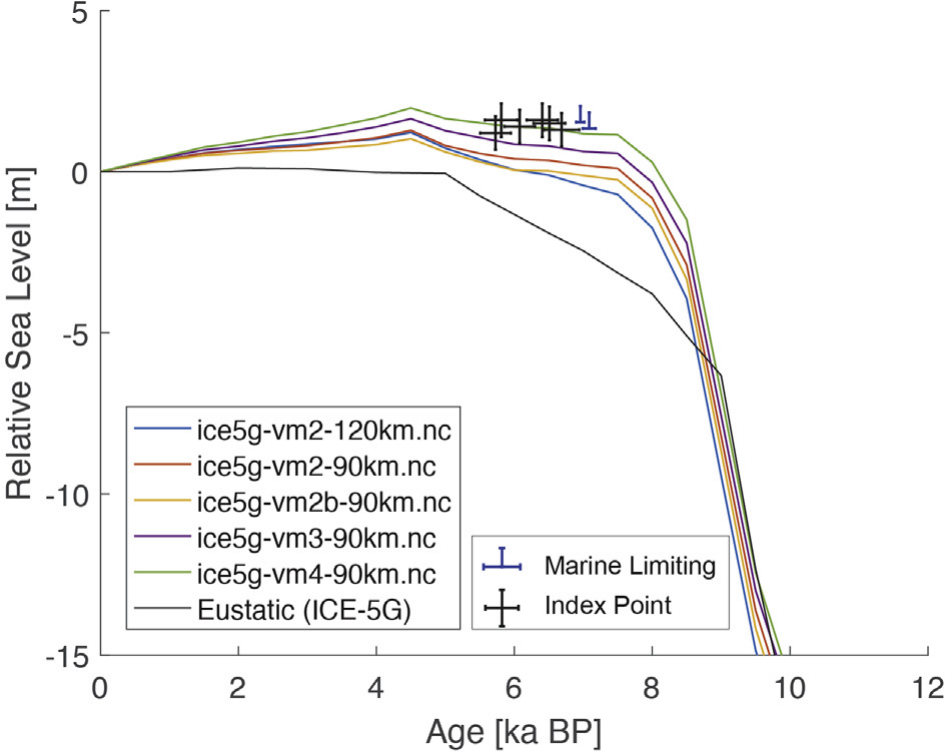
Standardized Holocene relative sea-level data obtained from Ref. [26] in comparison to glacial isostatic adjustment geophysical model predictions for Teluk Awur, Indonesia.

## 2 Experimental design, materials and methods

### 2.1 Relative sea-level data

The methods that have been applied to compile a standardized dataset of sea-level index and limiting points meet the criteria recently summarized by Ref. [2]. In those sites where the elevation of the Pleistocene unconformity relative to the analyzed sequence of Holocene relative sea-level data is known [i.e., Refs. 4, 5, 6], the tectonic overprint resulting from active uplift or long-term subsidence has been calculated. First, the average uplift/subsidence rate U has been calculated at each site. In doing so, minimum and maximum rates have been determined by dividing the minimum/maximum vertical displacements (based on the actual position of the Pleistocene Reef and a Last Interglacial sea level between 6 and 9 m above present) by the minimum/maximum time elapsed (based on a Last Interglacial between 116 ka BP and 129 ka BP). The average rate U is the sum of the minimum and maximum rates divided by 2 (negative rate for uplift, positive rate for subsidence). Calculated rates U are 0.18 m/ka for South Maalhosmadulu Atoll, Maldives [4], 0.19 m/ka for Palau Islands in the western Pacific [5] and −1.79 m/ka for Huon Peninsula, Papua New Guinea [6]. The corrected relative sea-level position at each site is then calculated as H + U × tc [following Ref. 6] where H is the actual sample elevation and tc the radiocarbon age of the sample. Details on the reconstructions of site-specific relative sea-level positions can be found in Ref. [1].

### 2.2 Glacial isostatic adjustment models

To compute the contribution of glacial isostatic adjustment to relative sea-level changes, we have solved the Sea Level Equation [27,28] by means of the SELEN program [29]. We employed a 1-D, radially stratified, self-gravitating, rotating, Maxwell viscoelastic and incompressible Earth model and the ice-sheet model ICE-5G [3]. To explore the sensitivity of the predictions to various aspects of the model, we employed different mantle viscosity profiles and lithosphere thicknesses (Table 1). All model runs include time varying coastline positions [3,30].
